# Invertebrate C1q Domain-Containing Proteins: Molecular Structure, Functional Properties and Biomedical Potential

**DOI:** 10.3390/md21110570

**Published:** 2023-10-30

**Authors:** Andrei Grinchenko, Ivan Buriak, Vadim Kumeiko

**Affiliations:** 1 School of Medicine and Life Sciences, Far Eastern Federal University, 690922 Vladivostok, Russia; grishagrin@mail.ru (A.G.); cutekasatik@gmail.com (I.B.); 2 A.V. Zhirmunsky National Scientific Center of Marine Biology, Far Eastern Branch, Russian Academy of Sciences, 690041 Vladivostok, Russia

**Keywords:** carbohydrate-binding proteins, lectin, C1q/TNF superfamily, cancer, clinical diagnostics, glycosylation, drug delivery

## Abstract

C1q domain-containing proteins (C1qDC proteins) unexpectedly turned out to be widespread molecules among a variety of invertebrates, despite their lack of an integral complement system. Despite the wide distribution in the genomes of various invertebrates, data on the structure and properties of the isolated and characterized C1qDC proteins, which belong to the C1q/TNF superfamily, are sporadic, although they hold great practical potential for the creation of new biotechnologies. This review not only summarizes the current data on the properties of already-isolated or bioengineered C1qDC proteins but also projects further strategies for their study and biomedical application. It has been shown that further broad study of the carbohydrate specificity of the proteins can provide great opportunities, since for many of them only interactions with pathogen-associated molecular patterns (PAMPs) was evaluated and their antimicrobial, antiviral, and fungicidal activities were studied. However, data on the properties of C1qDC proteins, which researchers originally discovered as lectins and therefore studied their fine carbohydrate specificity and antitumor activity, intriguingly show the great potential of this family of proteins for the creation of targeted drug delivery systems, vaccines, and clinical assays for the differential diagnosis of cancer. The ability of invertebrate C1qDC proteins to recognize patterns of aberrant glycosylation of human cell surfaces and interact with mammalian immunoglobulins indicates the great biomedical potential of these molecules.

## 1. Introduction

The complement system, as one of the most important molecular pathways of innate immunity, appeared only in vertebrates and humans, but the molecular evolution of the domains that formed its basis has occurred since the development of the first multicellular animals. One of the most important domains of the complement system proteins is the C1q domain, the evolution and spread of which has led to a wide variety of proteins of this group, generally referred to as C1q-domain-containing (C1qDC) proteins. In the absence of a complete complement system in invertebrates, the natural functions of the C1q/TNF superfamily proteins in these organisms are not fully understood. Evidence from recent decades suggests their involvement in molecular recognition mechanisms ranging from the early development of animals to the formation of specific immune responses, including the binding and destruction of pathogens. The involvement of C1qDC proteins in the mechanisms of molecular recognition has been repeatedly revealed before in the binding of PAMPs, the main motifs of which are formed by carbohydrate-containing biopolymers of pathogen cell surfaces. In this context, the discovery of proteins of this group among invertebrates often led to their identification as lectins, which previously included all carbohydrate-binding proteins.

Due to the amazing structural diversity of possible oligosaccharide motifs and the functionality of proteins recognizing them, non-covalent protein–carbohydrate interactions provide signaling at all levels from intracellular to inter-organisms. These interactions are especially important in cell–cell and cell–extracellular matrix communications, which underlie such processes as embryonic development, histogenesis, symbiogenesis, homeostasis, including immune responses, and many others. The most extensive and studied group of carbohydrate-binding proteins are lectins, which for a long time were considered the only ones with such functionality. Lectins include many families, each member of which had a structurally characteristic carbohydrate-recognizing domain (CRD) [1,2]. The wide representation, diversity, and characteristic functionality have led to the active use of lectins in various fields of biotechnology and biomedicine [3,4,5,6,7,8,9,10,11,12,13,14,15,16,17,18].

With the development of molecular analysis tools and advances in genomic research, many other carbohydrate-binding proteins have been identified [2,19,20]. One such group was the C1q domain-containing proteins, which are abundant in various invertebrates. C1qDC proteins and their genes and transcripts have been found in cnidarians, echinoderms, hemichordates, tunicats, nemerteans, annelids, rotifers, brachiopods, mollusks, and arthropods [21,22,23,24,25,26]. A particularly large number of C1qDC genes were found in the genomes of bivalves: 98 in *Crassostrea hongkongensis* (*Magallana hongkongensis*) [27], 296 in *Pinctada fucata* [28], 337 in *Crassostrea gigas* (*Magallana gigas*) [29], 408 in *Mercenaria mercenaria* [30], 445 in *Modiolus philippinarum* [31], 524 in *Mytilus edulis* [32], 554 in *Saccostrea glomerata* [33], 476 in *Crassostrea virginica* [34], 1182 in *Ruditapes philippinarum* [35], and more than 150 as transcripts in hemocytes of *Mytilus galloprovincialis* [36,37]. Probably such a huge number of genes arose as a result of multiple duplications of their genomic fragments. The pangenomics indicates the independence of these processes in different species [35]. The authors suggest that the abundance of C1qDC proteins in bivalves increases their protective potential against various pathogens due to the structural diversity of PAMPs that these proteins can bind. This provides a tangible advantage for adaptation to an environment saturated with microorganisms [23,29,35]. Due to the abundance of C1qDC genes in invertebrate genomes and a lack of their verified functional analysis, this review mainly considers isolated and somehow characterized C1qDC proteins. In addition, the bioinformatic tools for detecting proteins in genomes and transcriptomes are not always accurate, and predicted proteins may not correspond to reality. Therefore, here, we consider the obtained and functionally described C1qDC proteins with a focus on their structural features, functional properties, and biomedical potential.

## 2. C1qDC Proteins’ Structures and Phylogeny

**Figure 1:**
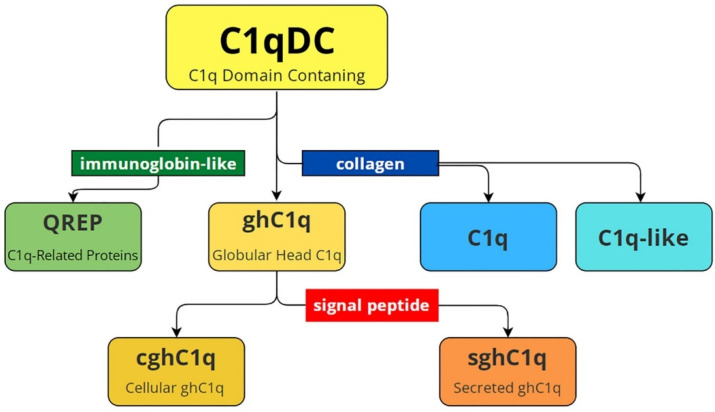
Structural variety of C1qDC proteins. C1qDC—C1q-domain-containing proteins; C1q-like—C1qDC proteins that have a collagen domain at the N terminus; C1q—the complement component 1q consisting of A, B and C polypeptide chains; QREP—C1q-related proteins with a single/several immunoglobulin-like domain(s) at the N-terminus; ghC1q—globular head C1qDC proteins; cghC1q—cellular globular head C1qDC proteins without signal peptide; sghC1q—secreted globular head C1qDC proteins with signal peptide.

One of the first reviews devoted to C1qDC proteins presents their structural classification [38], and this remains relevant with only one exception, which is modified by us in accordance with recent research (Figure 1). In general, C1qDC proteins are divided into globular head C1q (ghC1q) and C1q-containing collagen domain. The last one includes C1q and C1q-like proteins. Globular head C1q are divided into cellular proteins (cghC1q) and secretory proteins (sghC1q), which contain a signal peptide. Recently, proteins with N-terminal immunoglobulin-like motifs and C-terminal C1q domains have been found in Lophotrochozoa. They were named C1q-related proteins (QREPs) and classified as a new subfamily of variable immunoglobulin-bearing lectins [24]. Database analysis showed that QREPs are present only in the gastropods Heterobranchia and Caenogastropoda. Moreover, in Heterobranchia, QREPs are few in number, non-polymorphic, and represented by a combination of a C1q domain with a single immunoglobin-like domain. In contrast, in Caenogastropoda, QREPs have undergone massive expansion and may include either one or several immunoglobin-like domains [24]. This new subfamily of C1qDC proteins was additionally included in the modified diagram presented in Figure 1.

Many invertebrate C1qDC proteins are secretory pathogen-recognizing receptors and are able to non-covalently bind carbohydrates, which is actually a functional feature of lectins. Due to this pronounced similarity, many invertebrate carbohydrate-binding proteins are still classified as either lectins or lectin-like proteins [13,39]. During the period of earlier studying agglutinins without analyzing their structure, many carbohydrate-binding proteins were automatically assigned to the group of lectins. Later, it was found that many of the sialic-acid-binding lectins (SABL or SBL) contain the C1q domain [40,41,42,43,44], i.e., they belong to C1qDC proteins and are also considered in this review.

Many experimental articles devoted to C1qDC proteins present their phylogenetic trees [25,36,40,41,43,45,46,47,48,49,50,51,52,53,54,55,56,57,58,59,60]. Phylogenetic trees do not reflect the actual evolution of the C1qDC family of proteins for several reasons. Since the amino acid sequences of C1qDC proteins of invertebrates are characterized by a low percentage identity with the closest homologues, it is difficult to find a suitable outgroup for rooting a phylogenetic tree; therefore, unrooted and midpoint-rooted trees are more common in experimental articles. However, unrooted trees do not allow us to trace the direction of evolution of organisms from a common ancestor, and the limitation of using the midpoint rooting method is the imbalance of trees, which is determined by different evolutionary rates in different groups of organisms. Almost all phylogenetic trees were inferenced using the neighbor-joining method without validating the topology of the phylogenetic tree using probabilistic evolutionary methods. Due to the above reasons, phylogenetic trees have low (<<70) bootstrap node support, which gives an ambiguous picture of C1qDC proteins clustering. Even the clustering of some proteins into invertebrate/vertebrate clades is not always done [36,48,59]. Along with this, an interesting finding is the independent clustering of mollusk SABL with CfC1qDC from *Chlamys farreri* and AiC1qDC-1 from *Argopecten irradians* [43,47], which suggests the study of scallop proteins for the binding of sialic acids and their derivatives. The high variability of primary sequences indicates a high intertaxonomic heterogeneity of C1qDC proteins; therefore, the evolution of proteins of this family in invertebrates should be considered separately.

**Figure 2:**
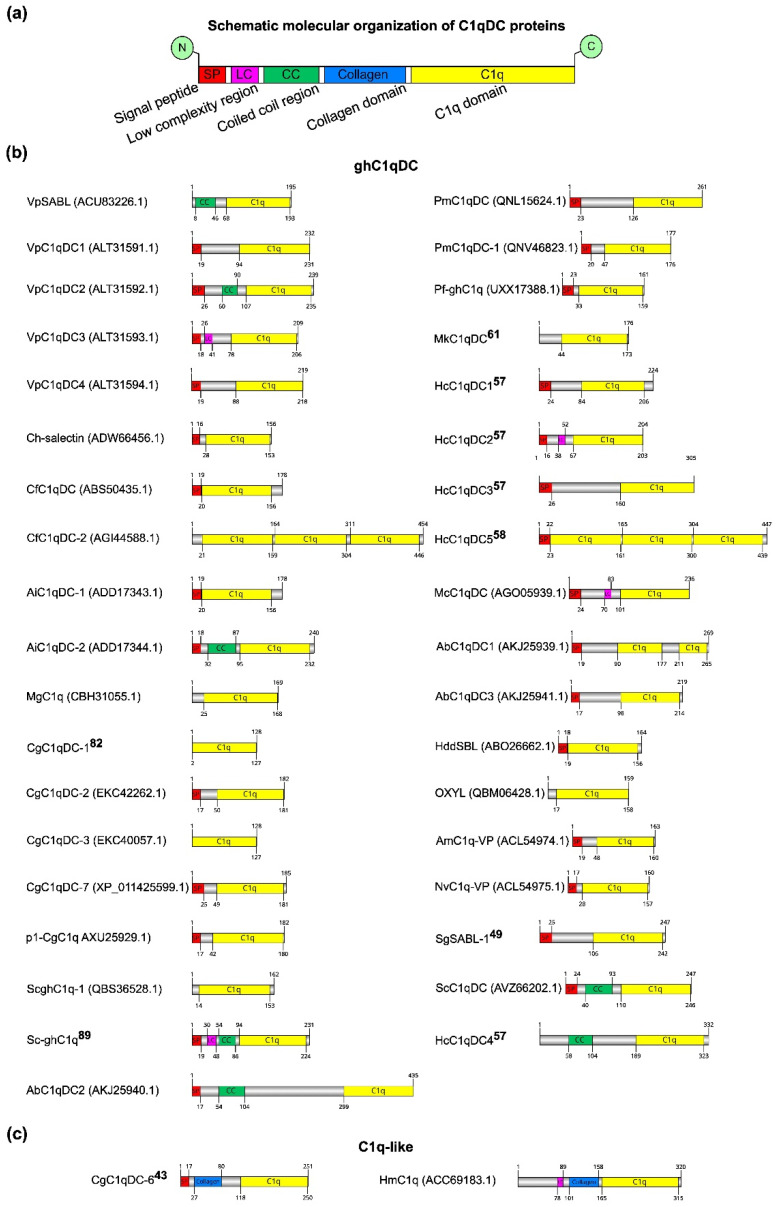
Domain organization of isolated C1qDC proteins. (**a**) Common domain architecture of C1qDC proteins; (**b**) list of globular head C1qDC structures; (**c**) list of C1qDC structures with collagen domain.

Most of the studied C1qDC proteins have been obtained in recombinant form. Only a few C1qDC proteins have been isolated and described in their native forms, such as SghC1qDC (OXYL) from the feather lily *Anneissia japonica* [25] and MkC1qDC recently isolated by us from the mussel *Modiolus kurilensis* [61]. The linear domain structure of C1qDC proteins was determined using SMART [62] (Figure 2). All analyzed C1qDC proteins have similar domain organization of functional elements. At the C-terminus of the C1qDC protein is the C1q domain, which occupies most of the primary sequence. The collagen domain serving for oligomerization is located before the C1q domain. At the N-terminus of secretory proteins are signal peptides required for extracellular transport. Of particular note is the coiled coil (CC) and low complexity (LR) regions located between the signal peptide and the collagen/C1q domain. The functional role of the CC and LR domains has not yet been established experimentally (Figure 2a). The length of the complete amino acid sequences ranges from 128 to 454 amino acids, and the length of the C1q domains ranges from 54 to 150 amino acids. Almost all C1qDC proteins have short signal peptides and one C1q domain. This classifies them as sghC1q proteins, which, probably, perform their functional role in a monomeric globular form (Figure 2b). The exceptions are the proteins VpSABL from *Venerupis philippinarum*, CfC1qDC-2 from *C. farreri*, MgC1q from *M. galloprovincialis*, CgC1qDC-1 from *C. gigas*, CgC1qDC-3 from *C. gigas*, ScghC1q-1 from *Sinonovacula constricta*, MkC1qDC from *M. kurilensis*, SghC1qDC from *A. japonica*, HmC1q from *Hirudo medicinalis*, and HcC1qDC4 from *Hyriopsis cumingii*. MkC1qDC from *M. kurilensis* and SghC1qDC from *A. japonica* do not contain the signal peptides since their amino acid sequences were obtained by mass spectrometry of the natively isolated proteins. Proteins VpSABL from *V. philippinarum*, MgC1q from *M. galloprovincialis*, CgC1qDC-1 from *C. gigas*, and CgC1qDC-3 from *C. gigas* do not contain the signal peptides, but they have been found to be involved in the immune response and expressed in the mantle, gills, and hemocytes, suggesting that they are secreted. The absence of signal peptides in proteins CfC1qDC-2 from *C. farreri*, ScghC1q-1 from *S. constricta*, HmC1q from *Hirudo medicinalis*, and HcC1qDC4 from *Hyriopsis cumingii* is due to the fact that they have intracellular localization or due to the inaccuracy of algorithms for predicting signal peptides in amino acid sequences. It is known that the increase in the length of the gene during evolution occurs mainly due to the duplication of domains. This mechanism may enhance protein functions by increasing the valency and avidity of its tertiary structure. Therefore, it is not surprising that among C1qDCs there are multidomain proteins. Thus, AbC1qDC1 from *Haliotis discus discus* includes two C1q domains, while CfC1qDC-2 from *C. farreri* and HcC1qDC5 from *H. cumingii* each contain three C1q domains. Proteins CgC1qDC-6 from *C. gigas* and HmC1q from *H. medicinalis* are C1q-like since they have collagen domains in the amino acid sequence necessary for protein oligomerization (Figure 2c). Interestingly, in the VpSABL from *V. philippinarum*, VpC1qDC2 from *V. philippinarum*, AiC1qDC-2 from *A. irradians*, Sc-ghC1q from *S. constricta*, ScC1qDC from *S. constricta*, HcC1qDC4 from *H. cumingii*, and AbC1qDC2 from *H. discus discus*, proteins were identified as coiled coil regions, not classified as collagen or collagen-like domains, which are supposed to play a similar structural role in the process of oligomeric protein formation. There are also VpC1qDC3 from *V. philippinarum*, Sc-ghC1q from *S. constricta*, HcC1qDC2 from *H. cumingii*, McC1qDC from *Mytilus coruscus*, and HmC1q from *H. medicinalis* proteins that contain so-called low complexity regions. For proteins with low complexity regions, a tendency to form amyloids has been shown [63], so it can be assumed that they can oligomerize through a self-assembly manner.

Currently, the crystal structures of important C1q domains from vertebrate C1qDC proteins have been described: C1q protein, adiponectin, cerebellin-1, caprin-2 [64,65,66,67], as well as several C1q-like vertebrate proteins were crystalized and investigated [68,69,70,71,72,73]. At the same time, the structural similarity of C1q with tumor necrosis factor (TNF) was initially noted, which was the reason for combining them into one C1q/TNF superfamily [65,74,75]. It is noteworthy that all the listed C1qDC proteins have different functions and diversified distribution in organisms. C1q protein is synthesized in the hematopoietic system and has various immune functions, including activation of the classical complement pathway [76,77,78]. Adiponectin is produced primarily in adipose tissue and has many functions, the main one being metabolic regulation [79,80]. Cerebellins are secreted adapter proteins that connect presynaptic receptors’ neurexins to postsynaptic ligands and thus participate in regulation and signaling in various brain structures [81]. Caprin-2 is an RNA-binding protein that enhances canonical Wnt signaling and functions in the central osmotic defense response, eye development, as well as tumor ferroptosis and metastasis [82,83,84,85]. Despite the variety of functions and sites of synthesis, the C1q domains in all cases have a characteristic jelly roll topology consisting of a ten-strand β-sandwich formed by two antiparallel five-strand sheets. At the same time, all of them form homo- or hetero-trimeric C1q domain structures mainly due to hydrophobic interactions. During the formation of trimer, a region appears for binding calcium ions, which also supports the formation of a trimeric structure, with the exception of cerebellin-1 [64,65,66,67].

Despite the absence of crystal structures of C1qDC proteins in invertebrates, their tertiary structures are modeled and compared using bioinformatics tools in some works. Even in a 2010 review, a high similarity of the tertiary structure of C1q domains was noted in phylogenetically very distant organisms, such as bacteria, mussels, and humans [38]. A characteristic jelly roll topology consisting of a ten-strand β-sandwich has been identified for CfC1qDC and CfC1qDC-2 from *C. farreri* [46,86], AiC1qDC-1 and AiC1qDC-2 from *A. irradians* [47,87], PmC1qDC from *P. fucata* [54], and BsC1qDC from *Botryllus schlosseri* [45], as well as an eight-strand β-sandwich for CgC1qDC-1 from *C. gigas* (*M. gigas*) [88]. At the same time, usually they show a low degree of identity with the amino acid sequences of human C1q domains, which were used as a reference [45,47,86,87]. However, in all cases, conservative aromatic amino acids involved in the formation of the hydrophobic part of the C1q domains, as well as some charged amino acids important for the structure, were identified [45,46,47,54,86,87,88].

**Figure 3:**
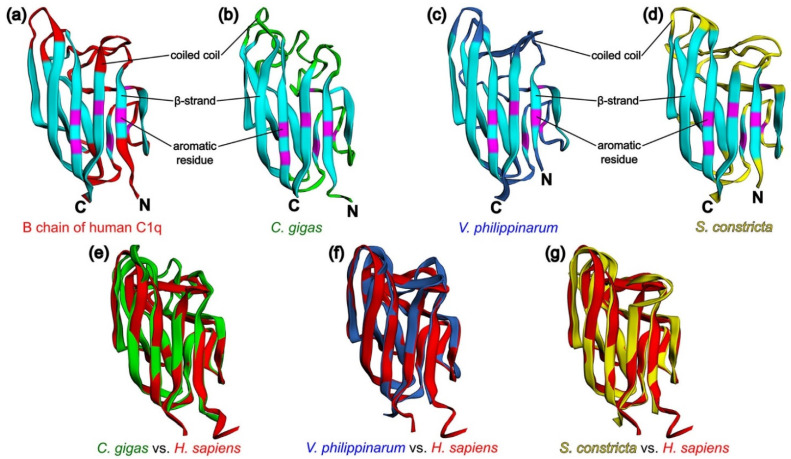
Structural comparison for three predicted C1qDC and their superposition with the B chain of human C1q. (**a**) Structural features of the B chain of human C1q (PDB code 1PK6) [64]: β-strands—blue, coiled coils—red, aromatic residues forming the hydrophobic core—purple; (**b**) homology model of p1-CgC1q from *C. gigas*; (**c**) homology model of VpC1qDC3 from *V. philippinarum*; (**d**) homology model of ScghC1q-1 from *S. constricta*; (**e**) superposition of p1-CgC1q from *C. gigas* (green cartoon) and the B chain of human C1q (red cartoon); (**f**) superposition of VpC1qDC3 from *V. philippinarum* (navy blue cartoon) and the B chain of human C1q (red cartoon); (**g**) superposition of ScghC1q-1 from *S. constricta* (yellow cartoon) and the B chain of human C1q (red cartoon).

To confirm the concept of similarity of the tertiary structures of C1qDC proteins, we modeled the structures of the p1-CgC1q from *C. gigas*, VpC1qDC3 from *V. philippinarum*, and ScghC1q-1 from *S. constricta* proteins by homology-based modeling server SWISS-MODEL [89] followed by their superposition full-atom structures on the modeled B chain of human C1q to determine the structural homology coefficients. It turned out that the modeled proteins have a typical jelly roll topology similar to the B chain of human C1q consisting of a ten-strand β-sandwich formed by two antiparallel five-strand sheets. Based on the modeling results, it becomes apparent that the conservative aromatic residues Phe97, Phe115, Tyr126, Phe133, Tyr141, Phe143, and Phe215 responsible for the formation of the hydrophobic core of the B chain of human C1q correspond to Phe51, Phe70, Tyr83, Phe90, Tyr98, Phe100, and Phe176 in p1-CgC1q from *C. gigas*, to Phe82, Phe101, Tyr112, Phe119, Tyr127, Phe129, and Phe201 in VpC1qDC3 from *V. philippinarum*, and to Phe23, Phe41, Tyr52, Phe59, Tyr67, Phe69, and Phe145 in ScghC1q-1 from *S. constricta*. As it appears, functional diversity of C1qDC proteins is ensured by variable coiled coil regions (Figure 3a–d). Comparisons of p1-CgC1q from *C. gigas*, VpC1qDC3 from *V. philippinarum*, and ScghC1q-1 from *S. constricta* with the B chain of human C1q using the TM-align method [90] were obtained, with TM values of 0.81018, 0.86206, and 0.84414, respectively (Figure 3e–g), which suggest generally the same fold of C1qDC proteins (TM-score > 0.5). EzMol web server was used to visualize the superposition of protein structures [91].

In general, the data point to a surprising similarity in the spatial organization of C1q domains with low identity of their primary sequences. Together with a simply organized domain structure including only one C1q domain for most isolated C1qDC proteins, as well as the ease of obtaining functionally active recombinant forms, these features make these proteins extremely attractive for protein engineering.

## 3. Biosynthesis and Tissue Distribution of C1qDC Proteins

Most of the data on the content of C1qDC proteins in tissues and organs of invertebrates were obtained by quantitative PCR. Among the most commonly found organs producing C1qDC proteins are hemocytes, digestive glands, muscles, mantle, gills, and gonads (Table 1). From the available data, it is impossible to identify a general pattern of C1qDC protein biosynthesis in tissues and organs: in each case, the distribution of transcriptional activity looks individual and does not depend on either the phylogeny of the species or the presence of immune function. However, the cells analyzed during immune challenge generally are hemocytes with an increase in the transcriptional activity after stimulation, even in the case of extremely low normal expression before immunogenic induction. The same goes for digestive glands, muscles, mantle, gills, or gonads in the cases where reverse transcription quantitative polymerase chain reaction (RT-qPCR) analysis was performed.

Quantitative PCR data do not provide insight into specific cellular sources other than hemocytes. Although, in the last case, the question of a specific cell type remains since different species include a different number of hemocyte populations. Animal organs contain many types of cells, each of which can potentially be a source of the studied proteins. In addition, all organs contain circulating cells, such as hemocytes, which can also be a source of C1qDC proteins, as noted earlier. More accurate in terms of cellular sources are in situ hybridization (ISH) and immunohistochemistry (IHC). However, only a few works have been performed using ISH or IHC assays. For example, PmC1qDC-1 from *P. fucata martensii* involved in immunity response and shell formation was found by fluorescence ISH (FISH) in gills (on gill rakers and gill filaments) [55] and mantle (mainly in the edge part) [96]. IHC of BsC1qDC from colonial ascidian *B. schlosseri* showed its presence in circulating phagocytes and morula cells with an increase during phagocytosis of fungi *S. cerevisiae* [45].

IHC analysis of SghC1qDC from *A. japonica* showed its presence in the regions surrounding the coelom and in spicules. These signals were overlapping with the DAPI signal, indicating that the lectin was produced by proliferating cells. Authors conclude that since SghC1qDC is a secretory protein that shows high solubility, its presence in such tissues can be explained even in the absence of its glycan ligands [25].

A detailed IHC analysis of the distribution of MkC1qDC was recently performed by us in the mussel *M. kurilensis*. The protein was detected in association with connective tissue fibers in mantle edge and digestive glands, in kidney concretions, as well as in interstitial space and the hemal system of all organs. Thus, the most intensively labeled organs were gills and pericardium with largest hemal sinuses and vessels [61]. The reason for such a distribution can be the same as in the SghC1qDC case because MkC1qDC is also a soluble protein. At the same time, intracellular localization was detected only in the granules of large hemocytes. Further analysis of their populations showed an abundant content of MkC1qDC in the granules of basophilic granulocytes, to a lesser extent in the granules of eosinophilic granulocytes, and also on the surface of agranulocytes [97].

## 4. Antibacterial Properties and Immune Functions of C1qDC Proteins

The antibacterial properties and involvement of C1qDC proteins in the immune response are tested using both PAMPs and microorganisms by several main approaches: protein binding to PAMP and lysates of microorganisms by immunoblotting; bacteria agglutination and its inhibition using PAMP; growth inhibition of microorganisms; testing the effect of C1qDC proteins on phagocytosis and chemotaxis; immune challenge with measuring the transcriptional activity of C1qDC protein genes by quantitative PCR.

The most commonly used microorganisms for immune challenges are *Vibrio* species, in particular, *Vibrio anguillarum* as well as *Vibrio splendidus*, *Vibrio alginolyticus*, and *Vibrio parahaemolyticus* (Table 2). Among Gram-positive bacteria, *Staphylococcus aureus* is often used for immune stimulation, but other species such as *Listeria monocytogenes*, *Micrococcus luteus*, and *Micrococcus lysodeikticus* can also be used. PAMPs stimulation is less common and limited by usage of LPS (lipopolysaccharide), PGN (peptidoglycan), GLU (glucan), and polyI:C (polyinosinic-polycytidylic acid). Fungi and viruses are rare in such studies for C1qDC proteins [45,47,87]. Quantitative PCR shows an increase in the transcriptional activity of C1qDC genes in almost all cases when induced by PAMP stimulation. Upregulation can range from a few times [40,45,51,56,57,58,60,92,94,95,96] to tens [36,41,43,52,55,59,88,93] and even hundreds of times [46,47,86,87] relative to control. Moreover, the response can occur both in a few hours and in a few days. For example, after the secondary challenge with *V. splendidus*, the upregulations of CgC1qDC-2 and CgC1qDC-4 mRNA in hemocytes occurred at 6 h, while that of CgC1qDC-3 was observed at 3 h and lasted for 24 h. CgC1qDC-3 responded with a high mRNA level for testing 24 h after the secondary challenge with *V. anguillarum* as well [48]. Hemocytes, as the main cells of the immune defense, were studied in all cases for the transcriptional activity of C1qDC genes during immune stimulation, even when the initial level of expression of these genes was minimal without stimuli [36,40,41,43,46,47,48,51,52,56,60,86,87,88,92,93,94,95,96]. However, the digestive glands, gills, mantle, and other organs are also analyzed [36,47,55,56,57,58,59,96]. A decrease in the transcriptional activity of C1qDC genes in response to stimulation is extremely rare and occurs only at certain time points after the challenge. For example, SgSABL-1 from *Solen grandis* had relatively low decreases in some time points after LPS, PGN, or GLU simulations [43], as well as HcC1qDC1 from *H. cumingii* after *S. aureus* or *Aeromonas hydrophila* challenges [57]. It is interesting to increase the transcriptional activity of C1qDC genes under the influence of pollutants, for example, oil products in the case of VpC1qDC1, VpC1qDC2, VpC1qDC3 and VpC1qDC4 from *V. philippinarum* (*R. philippinarum*) or heavy metals in the case of McC1qDC from *M. coruscus* (*Mytilus unguiculatus*), which makes C1qDC a potential tool for pollution monitoring [52,60]. It should be noted that an increase in transcriptional activity under the influence of pollutants does not always occur. Thus, in response to okadaic acid, the expression of complement C1q tumor necrosis factor-related protein 2 decreased in the gills of *A. irradians* [98]. When exposed to polychlorinated biphenyl Aroclor 1254 in the digestive gland of *Meretrix meretrix*, a decrease in the expression of C1q transcripts was found [99]. In addition, under the influence of CuO and Cu^2+^, a decrease in the differentially expressed putative C1qDC in the gills of *M. galloprovincialis* was observed [100]. The above data indicate that organic and inorganic pollutants can directly or indirectly inhibit the C1q protocomplement system, indicating a decrease in immune potential.

However, the analysis of transcriptional activity does not provide a clear understanding of the direct functionality of the studied protein. Since C1qDC proteins are considered as mainly pattern recognition receptors (PRRs), one of the main methods for demonstrating their involvement in interaction with pathogens is the analysis of the protein binding to PAMPs and/or microorganisms, usually by immunoblotting [48,49,50,51,57,58,88] or enzyme-linked immunosorbent assay (ELISA) [49,50,58,86,87,93]. The majority of studied C1qDC proteins from the oyster *C. gigas* (p1CgClq, CgC1qDC-1, CgC1qDC-2, CgC1qDC-3, CgC1qDC-4, CgC1qDC-5) have exceptional or extremely high specificity for Gram-negative bacteria and LPS [48,51,88,93], although CgC1qDC-6 and CgC1qDC-7 have a broader specificity, including LPS, PGN, MAN (mannan), polyI:C, fungi, and Gram-negative and Gram-positive bacteria [49,50]. All the studied C1qDC proteins from *H. cumingii* showed broad specificity for various bacteria, and all three C1q domains of HcC1qDC5 were able to bind LPS and PGN [57,58]. Moreover, AiC1qDC-2 from *A. irradians* and CfC1qDC-2 from *C. farreri* could bind different PAMPs, such as LPS, PGN, GLU, LTA, MAN, and polyI:C [86,87].

The fact of binding to pathogens and their components is not fully indicative of the functional role of the protein. Therefore, the ability to agglutinate microorganisms [25,47,51,53,61,93,94,95], inhibit their growth [25,41,55,61], and enhance phagocytosis and chemotaxis [46,48,49,50,56,88,93,95] is often additionally assessed. SghC1qDC from *A. japonica* was tested only with *P. aeruginosa*, whose agglutination is inhibited by the co-presence of N-acetyllactosamine, but not lactose or LPS. At the same time, SghC1qDC inhibited biofilm formation in *P. aeruginosa*, even though it did not affect bacterial growth [25]. ScghC1q-1 from *S. constricta* agglutinates both Gram-positive (*Bacillus subtilis* and *S. aureus*) and Gram-negative (*Escherichia coli* and *V. anguillarum*) bacteria [94], as well as MkC1qDC from *M. kurilensis* agglutinates, and inhibits the growth of *S. aureus*, *B. subtilis*, *Ruegeria* sp., *E. coli*, *Pseudoalteromonas* sp., and to a lesser extent *Vibrio* sp. [61]. In addition, growth inhibition of both Gram-positive and Gram-negative bacteria was shown for Ch-salectin from *C. hongkongensis* (*Bacillus thuringiensis*, *S. aureus*, *V. alginolyticus*, *E. coli*) [41] and PmC1qDC-1 from *P. fucata* (*Pseudomonas aeruginosa*, *B. subtilis*, *S. aureus*, *V. parahaemolyticus*, *A. hydrophila*, *E. coli*) [55]. Other C1qDC proteins have narrower specificity and/or functionality. For example, Sc-ghC1q and ScC1qDC from *S. constricta* agglutinate both Gram-positive and Gram-negative bacteria, but the last one is more intensive [53,95]. AiC1qDC-1 from *A. irradians* does not display obvious agglutination activity against *M. luteus* and *L. anguillarum*, but its mRNA production is stimulated by them as well as by the fungus *P. pastoris*, which is agglutinated by the protein very effectively, and in all cases AiC1qDC-1-specific expression was found in the digestive glands and hemocytes after different times of challenge [47]. CfC1qDC from *C. farreri* displayed a significantly strong activity to bind LPS from *E. coli*, although no obvious antibacterial or agglutinating activity toward *E. coli*, *L. anguillarum*, and *M. luteus* was observed, indicating the functionality of CfC1qDC only as a PRR [92]. In addition, in some cases, the studied C1qDC genes were knocked down with an assessment of functional changes during the immune response. The RNAi knockdown of p1-CgC1q in *C. gigas* hemocytes shows a significant decrease in both phagocytic activity and phagocyte fraction for in vitro phagocytosis [51]. A similar result was obtained for the knockdown of Pf-ghC1q in *P. fucata* [56]. RNAi of the BsC1qDC transcript from the colonial ascidian *B. schlosseri* reduced the activity of phagocytosis and the number of degranulated morular cells [45]. In the case of *H. cumingii*, RNAi results showed that HcC1qDC5 was involved in *V. parahaemolyticus*-induced HcTNF and HcWAP expression [58].

The C1q complex is the first component of the classical pathway of the complement system in vertebrates, initiating its activation upon interaction with antigen-bound IgG and IgM. The existence of a protocomplement system or its analogue in invertebrates has been actively discussed in recent years. In addition to C1q-like and C1qDC proteins, homologues of C2 and C3 of vertebrate complement proteins have also been found [101,102]. In this regard, mollusks stand out, especially bivalves. They also have homologues of C2 and C3 proteins [103,104,105,106,107,108], and many Bivalvia have a particular genetic diversity of C1q-like coding elements [28,29,31,33,34,35,36,37]. A variety of Bivalvia opsonins, including C1qDC proteins, can be effective substituted for antibodies, mannan-binding lectins, and ficolins that initiate the complement cascade in vertebrates [23]. In addition, some of the invertebrate C1qDC proteins are able to bind mammalian IgG and IgM [46,86,94], which additionally can be useful for biomedical applications.

All the presented data indicate the involvement of C1qDC proteins in the immune response as PRRs capable of binding PAMPs of different nature and structure to varying degrees. At the same time, functionally soluble C1qDC proteins mainly play the roles of agglutinins and opsonins, but they can also be involved in immune signaling.

## 5. Other Functions of C1qDC Proteins

Some works showed the transcriptional activity of C1qDC protein genes in embryonic development. In scallop *C. farreri* the mRNA transcripts of CfC1qDC and CfC1qDC-2 were detected in all investigated stages, and the expression level was up-regulated from D-hinged larva and reached the highest at eyespot larva [46,86]. The expression of MgC1q was detected all along the mussel *M. galloprovincialis* ontogeny, being detectable within 2 h post-fertilization, with a notable increase after 1 month and continuing to increase until 3 months [36]. In *P. fucata martensii*, PmC1qDC-1 expression was significantly high in the blastula and gastrula and especially high in the juvenile stage, which is the most important stage of dissoconch shell formation [96]. The transcription is modulated during the colonial ascidian *B. schlosseri* blastogenetic cycle, increasing during takeover events [45]. None of the papers presented provide insight into the specific role of C1qDC proteins in embryonic development, other than being of exceptional survival importance in the last example.

An exception is PmC1qDC-1, for which the authors attribute high transcriptional activity at key stages of shell formation. This suggestion is related to a more detailed study of PmC1qDC-1 in terms of its effect on the formation of the shell and its recovery after notching [54,96]. Notably, a group of proteins called KEYSTONEin from *Mytilus californianus* and *M. galloprovincialis*, in addition to a similar role in shell formation, has also been shown to play a role as a chemoattractant for predatory starfish, which extends the C1qDC value to an interspecies relation [109]. Another homologous C1q gene of the mollusk *P. fucata* (*P. fucata martensii*) PFMG4 also shows high expression in the mantle and participation in shell formation. After transfection of PFMG4 into mouse osteoblasts, their proliferation decreases with an increase in the production of type-I collagen, followed by biomineralization. The authors concluded that PFMG4 has potential ability in enhancing osteoblast differentiation, suggesting a new idea in developing therapeutics for the treatment of osteoporosis [110]. In addition, it was found that the C1qDC protein from *M. coruscus* (*M. unguiculatus*) provides stiffness to the byssus filaments through polymerization of the collagen-like region in the functional C1q domain with cysteine residues in protein sequence in a similar manner to collagen-like proteins [111].

Among other invertebrates, the involvement of *H. medicinalis* HmC1q protein in microglia activation upon nerve injury is of interest [112,113]. Moreover, it was shown that leech microglial cells also react to human C1q by chemotaxis assays, which indicates their similarity and probably biomedical potential. Another interesting example is C1q-VPs from *Apis mellifera* and *Nasonia vitripennis* (AmC1q-VP and NvC1q-VP, respectively), which are major polypeptides in their venoms and probably serve as toxin transporters. At the same time, AmC1q-VP is actively transcribed in most organs, not only in the venom gland [21].

Thus, the functions of invertebrate C1qDC proteins are not limited to immune surveillance (Table 3). An important and at the same time poorly elucidated aspect is their signaling role in embryonic development [36,45,46,86,96]. In addition, certain species have C1qDC proteins with very specific functions. Due to the low level of knowledge and the small number of isolated and described proteins of this group, there is a high probability that invertebrates have C1qDC proteins with previously undescribed functions and properties that may have biotechnological and biomedical potential.

## 6. Carbohydrate Specificity of C1qDC Proteins

The description of the carbohydrate specificity of C1qDC proteins is extremely rare: in addition to PAMPs, among all reviewed works, only two included a detailed assessment of the binding of the studied proteins to carbohydrates of various structures. The first was isolated from the crinoid feather star *A. japonica* and named SghC1qDC, or SghC1qDC [25,114]. Monosaccharides and disaccharides D-Gal, D-GalNAc, D-Glc, D-GlcNAc, D-Man, L-Fuc, sucrose, melibiose, and lactose did not inhibit hemagglutination even at 100 mM concentration. Fetuin, asialofetuin, porcine stomach mucin, and bovine submaxillary mucin showed inhibitory effects at concentrations of 0.1 mg/mL, 0.2 mg/mL, 0.4 mg/mL and 1.0 mg/mL, respectively. Affinity chromatography showed strong specificity to type-2 N-acetyllactosamine (LacNAc: Galβ1-4GlcNAc), but not to type-1 LacNAc (Galβ1-3GlcNAc). At the same time, the specificity for branched oligosaccharides increased with an increase in the number of Galβ1-4GlcNAc branches. In addition, SghC1qDC recognized type-2N-acetyllactosamine chains masked at the C-3 position of Gal by NeuAcα2-3; however, the protein did not recognize chains where the C-6 position of Gal had been masked by Neu5Acα2-6 [114].

Another example is MkC1qDC from the bivalve *M. kurilensis* that was isolated and substantially described in our laboratory [61]. Similar to most C1qDC proteins, MkC1qDC bound PAMPs (LPS, PGN, MAN), however, it showed the highest specificity for polysaccharides saturated with acidic galactans and mannans: alginate, κ-carrageenan, fucoidan, and pectin. Mono- and disaccharides such as D-Gal, 2-deoxy-D-Gal, L-Gul, D-GalA, D-GlcA, D-lactose, 2α-mannobiose, and Neu5Ac also inhibited hemagglutination with MkC1qDC but at significantly higher concentrations. Among simple sugars, MkC1qDC showed the highest specificity for sialic acid (Neu5Ac). As noted above, many other C1qDC proteins were previously attributed to sialic-binding lectins, for example, HddSLB, VpSABL, SgSABL-1, and Ch-salectin [40,41,42,43,44]. This highlights some similarities in their carbohydrate specificity spectrum. In contrast, the strong differences between SghC1qDC and MkC1qDC show a wide variability in the recognized glycans of C1qDC proteins.

The chapter Antibacterial Properties and Immune Functions of this review contains many examples of the binding of C1qDC proteins to PAMPs. It is significant that most of the used PAMPs have carbohydrates in their structures, which are ligands of C1qDC proteins [20,115,116]. At the same time, the carbohydrate components of PAMPs, in the presence of conserved regions, also have extremely variable elements, which largely affect their antigenic properties and recognition by the host [117,118,119,120,121,122]. Previously, it was noted that the investigated C1qDC proteins have different degrees of selectivity for both microorganisms and PAMPs, which is most likely due to the peculiarities of their carbohydrate-recognition repertoires.

Thus, C1qDC proteins as carbohydrate-binding PRRs with varying degrees of selectivity to pathogens and their PAMPs are extremely poorly studied in terms of carbohydrate specificity. Together with their wide distribution in invertebrate genomes and structural diversity (low homology), C1qDC proteins become extremely promising for studying their carbohydrate-binding properties and further usage as tools for glycobiology.

## 7. Biomedical Applications of Invertebrate C1qDC Proteins

Protein–carbohydrate interactions underlie many of the most important biological processes since they largely provide cell–cell and cell–extracellular matrix interactions. The most extensive and currently studied group of carbohydrate-binding proteins are lectins. However, recent intensive research in invertebrate biochemistry and genomics has led to the discovery of new groups of lectin-like molecules with similar properties, in particular, C1qDC proteins. The widest representation and characteristic functionality of lectins led to their active use in biotechnology, which began with ricin and arbin, found and isolated more than 100 years ago. At the moment, there are many reviews devoted to certain aspects of the use of lectins as tools in biomedicine and biotechnology [3,4,5,6,7,8,9,10,11,12,13,14,15,16,17,18,123].

Different carbohydrate binding proteins traditionally recognized as lectins still remain important tools in immunohematology, continuing to be classically used to detect specific erythrocyte antigens and activate various types of lymphocytes. In addition, the prospects of their use as tools for detecting stem cells through carbohydrate markers began to be discussed [4,8]. Their use in glycan mapping in histochemistry and cell biology also remains relevant [5]. The introduction of lectins into the now classic technology of enzyme-linked immunosorbent assay (ELISA) led to the emergence of enzyme-linked lectin sorbent assay (ELLSA), which differs in its focus on the detection of carbohydrate components [16]. Modern analytical methods using lectins also include lectin affinity chromatography, lectin blotting, analysis on microplates, microarrays, and biosensor technologies [10]. The enormous potential of this group of proteins and the current trend towards miniaturization of analysis technologies, both in science and in clinical practice, have led to an active discussion of the use of lectins in lab-on-a-chip systems, where the main idea is performing complex procedures using minimum analytes on microarray in one step [17].

The use of lectins in the composition of biocomposite materials and structures of various purposes and types is also actively developing, from glycosylated organic macrostructures to glyconanoparticles or glycan-bearing nanosystems based on inorganic matrices [3,124,125]. At the same time, a feature of using nanoparticles in the composition is their targeting by carbohydrate determinants, which can be used in drug delivery and oral immunization [9].

Many of the lectins show antiviral activity, and some of them have been put forward as candidates for the development of methods for the prevention or treatment of viral infections [6]. Potential targets include HIV, hepatitis, influenza, encephalitis, coronavirus, herpes simplex virus, and others [12]. At the same time, special attention is paid to research on the possibility of using lectins in the fight against AIDS and concomitant infections in HIV-infected people [11].

The problem of resistance to antibiotics in infectious bacteria and the difficulties in finding new effective antibiotics stimulate the search for new antimicrobial agents. Lectins are considered one of the promising groups in this research area, since many of them have immunomodulatory activity, including by triggering cytokine cascades and accelerating phagocytosis, and are also capable of directly destroying these pathogens [7,123]. In addition, the possibility of using vaccines based on complexes of heat-inactivated bacteria with lectins, which should cause active production of antibodies with a more diverse repertoire of recognizable antigenic determinants, is being considered [123], as well as the use of lectins as antifungal agents [7]. C1qDC proteins, which are widely distributed in invertebrates and exhibit a pronounced ability to bind both various microorganisms and components of their cell walls, also have significant potential as antimicrobial agents.

Since malignant transformation is closely associated with changes in the carbohydrate repertoire of the cell surface and extracellular matrix, carbohydrate-binding proteins can be a valuable tool for tumor diagnosis and potentially be used as part of therapeutic agents. For a number of lectins, cytotoxic effects have already been shown, manifested mainly due to the induction of apoptotic and autophagic pathways in malignant cells [13,18]. At the same time, options are considered that include their use in nanotheranostics, which is a combination of diagnostic and therapeutic functions in a single system based on nanotechnology [15]. In addition, changes in the structure and functioning of galectins on the surface of a number of transformed cells are known, which led to the development of possible treatment strategies based on the effect of glycomimetics and neoglycoconjugates on tumor galectins [14].

Cell surface sialylation is one of the most common and long-established aberrant glycosylations in oncogenesis and metastasis [126,127]. MkC1qDC from the mussel *M. kurilensis* showed significant specificity for sialic acid and mucin, which contain it. At the same time, MkC1qDC suppressed the growth of cervical adenocarcinoma cell line HeLa [61]. A number of other Bivalvia C1qDC proteins are also sialic-acid-specific [40,41,43], as well as HddSLB from the gastropod *H. discus discus*. HddSLB has been tested on several cell lines and exhibited pronounced antitumor activity [42,128,129]. In particular, tumors of epithelial origin such as hepatocellular carcinoma Hep3B, lung cancer A549, non-small cell lung cancer H1299, colorectal adenocarcinoma SW480, as well as leukemia K562/ADR and glioma U87MG were sensitive [128,129]. In addition, HddSLB reduced the adverse effects of thymidine kinase-deficient oncolytic vaccinia virus on in vivo mouse models with subcutaneously transplanted C6 rat glioma cells and significantly increased animal survival [129].

LacNAc-specific C1qDC protein from crinoid feather star *A. japonica* SghC1qDC binds to the cell surface of breast tumor lines BT-474, MCF-7, and T47D, as well as cervical tumor cell line HeLa. Complete inhibition of binding by LacNAc did not occur in the case of the MCF-7 and HeLa lines, which may be due to overexpression of LacNAc containing glycans on their surface or due to the presence of other carbohydrate ligands. At the same time, SghC1qDC did not pass into the cells and did not show obvious cytotoxicity even after prolonged incubation up to 12 h [25].

**Figure 4:**
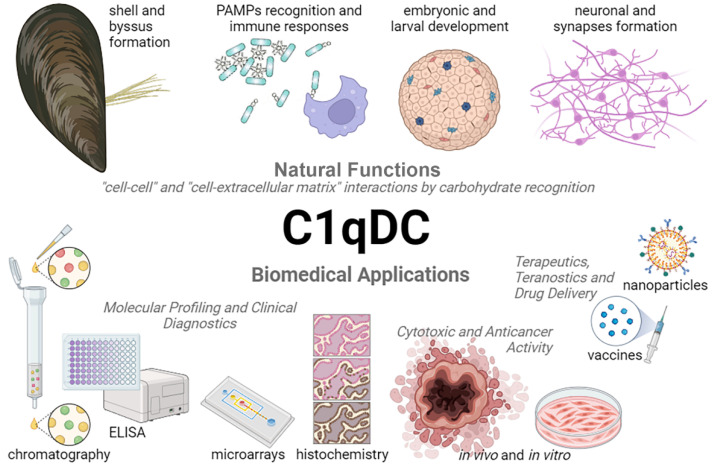
Natural functions and biomedical applications of C1qDC proteins.

Invertebrate C1qDC proteins are common carbohydrate recognition receptors, which bind the pathogens via PAMPs [20]. The main part of PAMPs are complex carbohydrates that are characterized by high structural heterogeneity. LPS and PGN are the most used PAMPs for evaluating the role of C1qDC proteins in the immune defense of invertebrates (Table 2). The immunomodulatory properties of bacterial LPSs are determined by the structural diversity of O-antigens. For example, *Klebsiella pneumoniae* O3a and *E. coli* O9a are characterized by the presence of high mannose repeat structure, while *Salmonella enterica* serovar Borreze O:54 has N-acetylmannosamine repeat residues [122]. The glycan chains of PGN are composed of repeating disaccharide structures of N-acetylglucosamine and N-acetylmuramic acid [121]. At the same time, glycosylation is the most complex and diverse post-translational modification in animal cells, including humans [130]. Aberrant protein glycosylation contributes to the development and progression of cancer, which allows us to consider altered glycosylation as a promising target for diagnosis and targeted therapy [131,132,133,134,135,136]. Since C1qDC proteins bind carbohydrate motifs in PAMPs, it can be assumed that they will recognize similar terminal glycosylation in cancer cells. The abundance of high mannose N-glycans characterizes the progression of breast cancer [137], prostate cancer [138], colorectal cancer [139], and cholangiocarcinoma [140]. Terminal β1,6-GlcNAc branching in N-glycans has been shown to be involved in cancer growth and metastasis [141,142]. Thus, C1qDC proteins can be considered as potential molecular tools for the detection and therapy of malignant cells with a specific glycosylation profile (Figure 4).

## 8. Conclusions

This review explored the potential of understudied C1q/TNF superfamily orthologues in invertebrates as tools for bioengineering and biomedical applications. Through a comprehensive analysis of the literature, gaps have been identified in this area of biological knowledge and a roadmap has been outlined for future research on hot spots in the biochemistry and physiology of C1qDC proteins. Despite the fact that C1qDC proteins are classical PAMP-recognizing molecules, little is known about their carbohydrate-binding properties, although the diversity of C1qDC proteins provides the coverage of the structural features of carbohydrate patterns in pathogens. The ubiquitous involvement of C1qDC proteins in various physiological processes makes this group of proteins attractive for the development of veterinary diagnostic assays for invertebrate pathology. Structural homology of these proteins with human C1q inspires the development of new immunotherapeutic agents and protein-based immunostimulatory adjuvants for vaccines targeted to diversified carbohydrates determinants of pathogens or malignant cells. The ability of invertebrate C1qDC proteins to recognize patterns of aberrant glycosylation of human cell surfaces and interact with mammalian immunoglobulins also indicates the great biomedical potential of these molecules. This review fosters further investigations of glycocode recognition by C1q homologues, identifying patterns of structure–activity relationships, as well as the development of new technologies for precise clinical diagnostics and modern strategies to combat cancer and infections.

## Figures and Tables

**Table 1:** Tissue distribution of C1qDC transcripts in different species determined by RT-qPCR (data are normalized to tissue with the lowest expression for each protein separately, i.e., comparison of expression rates between different proteins is not implied).

Species	Protein	Hm	Dg	Ms	Mn	Gl	Gn	Other	References
*V. philippinarum* *(R. philippinarum)*	VpSABL	1	40	1	160	20			[40]
*S. grandis*	SgSABL-1	1	221	1	2	209	3		[43]
*C. hongkongensis* *(M. hongkongensis)*	Ch-salectin	1	2	3	1	6	6	heart: 2	[41]
*C. farreri*	CfC1qDC	1		16	16	16		kidney: 23	[92]
	CfC1qDC-2	1	5972	67	46	12	425	kidney: 62	[86]
*A. irradians*	AiC1qDC-1	1	5888	20	8	4		heart: 14	[47]
	AiC1qDC-2		483	1	51	22	78		[87]
*M. galloprovincialis*	MgC1q	4000	2	1	1	2	450		[36]
*M. coruscus* *(M. unguiculatus)*	McC1qDC	670	1	1	3	7	4	foot: 1	[60]
*C. gigas* *(M. gigas)*	CgC1qDC-1	81	4	1	82	3	2		[88]
	CgC1qDC-2	70	38	58	0	5	1		[48]
	CgC1qDC-3	20	3	8	260	45	1		
	CgC1qDC-4	10	1	1	30	1	11		
	CgC1qDC-5	15	1	1	13	1	21		[93]
	CgC1qDC-6	7	1	4	4	1	1		[49]
	CgC1qDC-7	8	1	1	2	5	2	labial palp: 1	[50]
	p1-CgC1q	70	80	10	20	10	20	heart: 1	[51]
*S. constricta*	ScC1qDC	4	1500			3		foot: 1 siphon: 4	[53]
	ScghC1q-1	8	58000		6	9	12	foot: 1 siphon: 10	[94]
	Sc-ghC1q	5	15000		2	4	17	foot: 2 siphon: 3	[95]
*P. fucata*	PmC1qDC		70	1	60	60		foot: 10	[54]
	PmC1qDC-1	1		2	4–47	4		foot: 15	[96]
	Pf-ghC1q	2	1	3	2	4			[56]
*H. cumingii*	HcC1qDC5	1	12		8	3			[58]
*H. discus discus*	AbC1qDC1	1	2100	150	100	5		digestive tract: 0	[59]
	AbC1qDC2	1	55	60	60	2		digestive tract: 1	
	AbC1qDC3	1	15	20	7	0		digestive tract: 4	

Hm—hemocytes; Dg—digestive glands; Ms—muscle; Mn—mantle; Gl—gill; Gn—gonad.

**Table 2:** Immune stimulators used to produce C1qDC proteins in different species.

Species	Proteins	PAMPs	G- Bacteria	G+ Bacteria	Other	References
*V. philippinarum* *(R. philippinarum)*	VpSABL		*V. anguillarum*			[40]
	VpC1qDC1 VpC1qDC2 VpC1qDC3 VpC1qDC4				*soluble fraction of No.0 diesel oil*	[52]
*S. grandis*	SgSABL-1	LPS PGN GLU				[43]
*C. hongkongensis* (*M. hongkongensis)*	Ch-salectin		* V. anguillarum *			[41]
*C. farreri*	CfC1qDC	LPS PGN GLU polyI:C	* Listonella * *anguillarum*			[46,92]
	CfC1qDC-2	LPS PGN GLU polyI:C				[86]
*A. irradians*	AiC1qDC-1		* L. anguillarum *	* M. luteus *	Fungi *Pichia pastoris*	[47,87]
*M. galloprovincialis*	MgC1q		* V. anguillarum *	*M. lysodeikticus*		[36]
*M. coruscus* *(M. unguiculatus)*	McC1qDC		*V. alginolyticus* *Vibrio harveyi*		*Cu^2+^* *Cd^2+^*	[60]
*C. gigas* *(M. gigas)*	CgC1qDC-1		* V. splendidus *			[88]
	CgC1qDC-2 CgC1qDC-3 CgC1qDC-4		*V. splendidus* *V. anguillarum*			[48]
	CgC1qDC-5	LPS	*V. splendidus* *V. anguillarum*			[93]
	p1-CgC1q		* V. alginolyticus *			[51]
*S. constricta*	ScghC1q-1		* V. anguillarum *	*S. aureus*		[94]
	Sc-ghC1q		* V. anguillarum *	*S. aureus*		[95]
*P. fucata*	PmC1qDC-1	LPS PGN polyI:C				[55,96]
	Pf-ghC1q		* V. alginolyticus *			[56]
*H. cumingii*	HcC1qDC1 HcC1qDC2 HcC1qDC3 HcC1qDC4 HcC1qDC5		* A. hydrophila *	*S. aureus*		[57,58]
*H. discus discus*	AbC1qDC1 AbC1qDC2 AbC1qDC3		* V. parahaemolyticus *	*L. monocytogenes*		[59]
*B. schlosseri*	BsC1qDC *			*Bacillus clausii*	Fungi *Saccharomyces* *cerevisiae*	[45]

* The protein was not obtained; the work was carried out only on the transcript.

**Table 3:** Not immune functions of C1qDC proteins in different species.

Species	Proteins	Functions or Involving Process	References
*C. farreri*	CfC1qDC CfC1qDC-2	embryonic development	[46] [86]
*M. galloprovincialis*	MgC1q	embryonic development	[36]
*B. schlosseri*	BsC1qDC	embryonic development	[45]
*P. fucata martensii*	PmC1qDC-1	embryonic development shell formation and recovery	[96] [54,96]
*M. californianus*, *M. galloprovincialis*	KEYSTONEin	shell formation and recovery chemoattractant for predatory starfish	[109]
*M. coruscus* *(M. unguiculatus)*	C1qDCs	byssus filaments formation	[111]
*H. medicinalis*	HmC1q	microglia activation and nerve system development	[112,113]
*A. mellifera* *N. vitripennis*	AmC1q-VP NvC1q-VP	toxin transporters	[21]

## Data Availability

Data sharing not applicable.
